# The running performance characteristics of U16 and U18 inter-county Ladies Gaelic Football players: An analysis across halves, playing positions, and age grades

**DOI:** 10.1371/journal.pone.0349785

**Published:** 2026-05-29

**Authors:** Teresa Molohan, Stephen Behan, Sarahjane Belton, David Nolan

**Affiliations:** 1 Faculty of Science and Health, School of Health and Human Performance, Dublin City University, Glasnevin, Dublin, Ireland; 2 Insight SFI Research Centre for Data Analytics, Dublin City University, Glasnevin, Dublin, Ireland; 3 Co-Ex|Lab, Faculty of Science and Health, School of Health and Human Performance, Dublin City University, Glasnevin, Dublin, Ireland; Technological University Dublin - Tallaght Campus, IRELAND

## Abstract

This study examined the running performance demands of U16 and U18 inter-county Ladies Gaelic Football match play across halves, playing positions, and age grades using 10 Hz portable GPS technology (Catapult One, Melbourne, Australia). A total of 113 inter-county players (U16: height 166 ± 5.5 cm, body mass 59.5 ± 6.8 kg; U18: height 168 ± 5.1 cm, body mass 63.0 ± 8.2 kg) participated in the study. Data from competitive championship matches were collected over two seasons from nine U16 and twelve U18 games, resulting in 76 (U16) and 107 (U18) player profiles, respectively. A mixed repeated measures multivariate analysis of variance (MANOVA) evaluated the effects of age grade, positional line (full-back, half-back, midfield, half-forward, full-forward), and half of play (1st vs. 2nd) on variables including: Total Distance (TD), High-Speed Running (HSR), Max Speed, Relative Distance (m·min^-1^), Distance in Speed Zones 4 and 5, Accelerations (≥ 3 m·s ⁻ ^2^), and Decelerations (≤ −3 m·s ⁻ ^2^). Multivariate analysis for within- and between-subjects factors showed significant main effects for halves of play (*p* < 0.001, η_p_^2^ = 0.431), team (*p* < 0.001, η_p_^2^ = 0.300), and playing position (*p* < 0.001, η_p_^2^ = 0.183) on the combined dependent variables. Results demonstrated a consistent decline in running performance across halves, regardless of position or age. The ‘middle eight’ positions demonstrated the highest match-play demands and a distinct running profile compared to ‘inside’ lines, while U18 players showed significantly higher total and high-speed running distances than U16 players. This research is the first to examine the running performance profiles of U16 and U18 inter-county LGF players and may aid coaching by providing reference data for talent identification, individual player monitoring, and guiding developmental progression within the player pathway.

## Introduction

Ladies’ Gaelic football, governed by the Ladies Gaelic Football Association (LGFA), is a major participation sport for females in Ireland [1–3]. Club participation begins with non-competitive “Go Games” (ages 8–12) at the local community level. Subsequently, players transition to competitive age-group structures from U13 through to adult grades [4,5]. There are two primary tiers of competition within the LGFA: club and inter-county, with inter-county competition considered the highest competitive standard. The best-performing young players are selected to represent their county at U14, U16, and U18 levels, and compete in provincial and national competitions as part of the player pathway [3]. These competitions serve as process markers of development, with the overarching objective of facilitating the transition of young players towards competition at the senior representative level [3]. Matches are played on a large rectangular pitch measuring 130–145 m in length and 80–90 m in width, with H-shaped goalposts at each end [2,4,6]. Each game lasts 60 minutes, split into two 30-minute halves. A team comprises 15 players organised across six positional roles: one goalkeeper, three full-backs, three half-backs, two midfielders, three half-forwards and three full-forwards [2,3,6–9]. Akin to other invasion games such as soccer, rugby and Australian Rules football, players attempt to outscore the opposition by maintaining possession to create scoring opportunities, while limiting the opposition’s attacking threats [2,6–8]. Kicking or palming the ball below the crossbar into the net results in a goal, worth three points, while sending the ball through the goalposts by hand or foot over the crossbar results in a point [6–8]. During these attacking and defensive situations, players are required to employ the skills of the game, including catching, handpassing, kicking, blocking, bouncing, soloing and tackling [2,7–9]. Locomotive movements in the game are multidirectional and intermittent, with lower-intensity efforts including walking, jogging, and standing punctuated by intervals of high-intensity running, sprinting, acceleration, deceleration, and changes of direction [4,6,9].

Global Positioning Systems (GPS) technology is now commonly used in team sports such as soccer, Australian Rules, Gaelic football, hurling, camogie, hockey, and both rugby codes [10–14]. GPS technology’s ability to simultaneously track the movement of multiple athletes is particularly useful in intermittent team sports, where running is the predominant physical activity and frequent changes in direction occur [15]. Analysis of the running demands includes total distance, maximal speed, relative distance, accelerations, decelerations, and distances covered in various speed zones (e.g., walking, jogging, running, high-speed running, and sprinting) [15–18]. Furthermore, recent advancements in sampling rates and firmware updates have improved GPS units’ validity, reliability, and interunit reliability [11,13,19,20]. This technology has enabled the physical demands of training and match play to be assessed, providing coaches with the necessary data to design training programmes that effectively replicate these demands [6,10,12,13,15].

Research on the running requirements of Ladies’ Gaelic football is sparse [1]. To date, only two studies have been published reporting the running demands at senior inter-county level [2,6]. These studies found that, independent of playing position, the average distance covered during match play was 7319 ± 1021 m, with an intensity level of 116 ± 9 m·min^-1^ [6]. High-speed running (≥ 4.4 m·s^−1^) accounted for 1547 ± 432 m of the total distance covered during the game, with a mean maximum velocity attained of 7.17 ± 0.41 m·s^−1^ [6]. Positional analysis found that the middle eight positions (half-backs, midfielders, and half-forwards) covered the greatest distances, with these positions covering significantly more distance than the full-back and full-forward lines [6]. Analysis of match-play by quarter revealed the highest running demands in the first quarter for all positional lines, followed by reductions in subsequent quarters [2]. Reductions in locomotion were consistently observed in the second and fourth quarters, with the most significant reductions noted in the middle eight positions [2]. These variations in locomotion and positional differences have been attributed to the technical and tactical requirements of match play and are similar to findings in men’s Gaelic football and hurling, as well as in other female team-sport invasion games such as soccer, Australian rules football, camogie, and hockey [10,13,14,18,21,22].

An important part of developing players for senior-level competition is ensuring that they can meet the game’s physical demands [23]. Analysis of the running requirements at senior inter-county level has broadened our understanding of the demands faced by players and teams at the highest level. However, it is limited in its consideration of the broader physical demands at non-elite levels and lacks alignment to development pathways designed to prepare players for the demands of the sport at senior level [24]. Previous research conducted in women’s rugby sevens, soccer, and AFLW have found that the demands of match play increase with age and playing level [24–28]. In rugby sevens, small to moderate differences were observed from junior to senior and elite levels in sprint distance, relative distance, and maximum speed [26]. In soccer, as players progressed from U15, U16, and U17 age grades through to collegiate, professional, and international levels, the total match distance increased linearly from 6,936 ± 335 m to 10,144 ± 546 m, while the intensity of play rose from 87 ± 4 m·min^-1^ to 111 ± 6 m·min^-1^. Younger players also spent proportionally more time walking and less time engaged in high-intensity running (≥ 4.4 m·s^-1^) compared to international players (545 ± 141 m vs. 1,251 ± 276 m) [24]. The greater demands through youth and into international levels were somewhat related to longer match durations (80 min vs 90 min), but more importantly, to higher match intensity [24]. Similarly, when comparing the match-play activity demands of the talent pathway with those of the AFLW, the most notable differences were the relative distance and very high-speed running demands. Players in the AFLW cover 117 m·min^−1^ on average, compared to 111 m·min^−1^ in the U18 national championship, while very high-speed running distances are reported to be two to three times higher (107–238 m) in the AFLW than those experienced in elite-junior (48–85 m) competition [27].

While the benchmarks derived from rugby, soccer, and AFLW provide valuable insights, comparable longitudinal tracking data remains severely limited across most other adolescent female field sports. Consequently, these sports provide the only available objective benchmarks for adolescent female athletes. This lack of comparative data across other female invasion-based games further highlights the need for the present study to establish sport-specific performance profiles to guide the LGF developmental pathway. Differences in playing numbers, pitch dimensions, rules, technical skills and tactics may not fully translate to the unique demands of Ladies Gaelic Football. To date, no studies have been published on the running requirements of U16 and U18 inter-county Ladies Gaelic football. Understanding the running demands at these levels will provide information regarding the physical requirements at each age grade [29]. This will enable coaches to make evidence-informed decisions about training design, volume, and intensity, ensuring that players are adequately prepared for the demands of inter-county competition while also considering their long-term development. Therefore, the current study aimed to examine the running performance demands of U16 and U18 inter-county match play across halves of play and between positions. It was hypothesised that running performance demands would decrease between halves at each age grade, and that the middle eight positions (half-backs, midfielders, and half-forwards) would achieve greater total distance (TD), high-speed running (HSR), and sprint distance than the full-back and full-forward lines. It was also hypothesised that U18 players would exhibit greater performance demands for TD, HSR, and sprint distance than U16 players.

## Methods

### Experimental approach to the problem‌‌

The current study was designed to examine the match-play running performance of U16 and U18 inter-county ladies’ Gaelic football players across halves of play using portable GPS technology. Both age grades were monitored in competitive championship play over a two-season period (18^th^ March 2023−11^th^ July 2024; U16, n = 9 matches; U18, n = 12 matches). Challenge games were excluded from the analysis. Individual samples were collected from players who completed the full 60 minutes, resulting in 76 (U16) and 107 (U18) player profiles, respectively. Data were classified based on positional line and across halves of play, yielding the following number of samples per playing position: U16 full back (FB) n = 20, half back (HB) n = 20, midfield (MF) n = 11, half forward (HF) n = 14, full forward (FF) n = 11; U18 full back (FB) n = 31, half back (HB) n = 27, midfield (MF) n = 11, half forward (HF) n = 21, full forward (FF) n = 17. Data were collected 1–6 times per player throughout the investigation period. All matches were played at 14:00 on weekend afternoons, except for three in the latter stages of the championship, which took place at 19:30 on weekdays. Temperatures during match play ranged from 6 to 15 °C. All players completed two pitch and one gym session per week during the observation period and were requested to refrain from strenuous physical activity in the 24 hours prior to competitive matches.

### Subjects

One hundred and thirteen (n = 113) inter-county U16 (height 166 ± 5.5 cm, body mass 59.5 ± 6.8 kg) and U18 (height 168 ± 5.1 cm, body mass 63.0 ± 8.2 kg) players participated in the study. The U16 and U18 squads comprised 15- and 16-year-old players and 17- and 18-year-old players, respectively. Ethical approval was granted by the Dublin City University Research Ethics Committee (DCUREC/2022/122), and written parental consent and player assent were obtained prior to sample collection.

### Experimental procedure

Players were equipped with individual GPS units containing a triaxial accelerometer and a sampling rate of 10 Hz to monitor their running performance during match play (Catapult One Pod; Catapult Sports; Melbourne, Australia). A specially designed sports vest was worn beneath the player’s jersey, with the GPS unit (84 mm × 42 mm × 21 mm) positioned between the scapulae in a protected pouch. Players used the same GPS device for all observations to mitigate inter-unit error [30]. Activation and satellite lock were established by turning the units on 15 minutes prior to the pre-match warm-up [10,18]. The validity and reliability of 10 Hz devices utilising the PlayerTek technology found in the Catapult One have previously been established and reported as acceptable across a range of velocities and distances in intermittent team sports [9,16,20,31,32]. Specifically, these units have demonstrated acceptable reliability for measuring total locomotive distances during shuttle-based movements, with coefficient of variation (CV) ranges of 2.2–7.1% for 10 m shuttle distances and 1.0–3.1% for 20 m shuttle distances [33]. Furthermore, the units demonstrated high inter-unit consistency during dynamic, sport-specific football activity (CV: 1.95–7.20%) [33]. Previous research in an elite inter-county Gaelic football context, utilising identical 10-Hz units, also confirmed acceptable reliability for key high-speed metrics, with CV’s of 5.8% for high-speed running distance (HSRD) and 11.7% for very high-speed running distance (VHSRD) [9]. GPS data were retrospectively downloaded after each competitive game using proprietary software (Catapult One Version 6.00.0000, Catapult Sports, Melbourne, Australia). The data file was trimmed to include only activity that occurred during each half of match play. Subsequently, the file was exported to a Microsoft Excel spreadsheet (Microsoft, Redmond, WA, USA). The Excel spreadsheet enabled categorisation of activity into total distance (TD) (m), high-speed running (HSR) (m; ≥ 4.4 m·s^-1^), sprint distance (m; ≥ 5.5 m·s^-1^), accelerations (n; ≥ 3 m·s^-2^), decelerations (n; ≤ −3 m·s^-2^), relative distance (m·min^−1^), and maximal speed (m·s^−1^). Zone 4 running was defined as speeds of 4.4–5.5 m·s^−1^, while zone 5 referred to sprint speeds of ≥ 5.5 m·s^−1^. HSR is defined as the total distance covered at ≥ 4.4 m·s^-1^ and thus includes sprint distance. An acceleration or deceleration was categorised once a player’s speed changed by 3 m·s^−2^ [30]. These specific speed thresholds were selected because they align with established age-grade and female invasion sports research, including ladies’ Gaelic Football [2,6,10,13,14,24,28,34], thereby facilitating standardised, objective tracking and comparison of developmental progression between the U16 and U18 cohorts.

### Statistical analysis

All statistical analyses were performed using SPSS for Windows (Version 29, SPSS Inc., Chicago, IL, USA). Descriptive data are presented as mean ± standard deviation (SD). Initial whole-match comparisons between the U16 and U18 age grades were conducted using independent-samples t-tests, with 95% confidence intervals (CIs) reported for the mean differences. Subsequently, a mixed repeated-measures multivariate analysis of variance (MANOVA) was employed to examine the effects of team (U16 vs U18) and positional line (full-back, half-back, midfield, half-forward, and full-forward) on multiple dependent variables measured across two time points (1st half vs 2nd half). The dependent variables included: TD (m), HSR (m), Max Speed (m·s^−1^), Distance Per Minute (m·min^−1^), Distance in Speed Zone 4 (m), Distance in Speed Zone 5 (m), Accelerations (≥ 3 m·s^-2^), and Decelerations (≤ −3 m·s^-2^). Levene’s Test of Equality was conducted for each dependent variable to assess between-subjects effects and the assumption of homogeneity of variance. Pillai’s Trace was used as the multivariate test statistic. Bonferroni-corrected post hoc pairwise comparisons were conducted to identify specific group differences when a significant interaction was observed. The significance level for all statistical tests was set at *p* < 0.05. Where significant effects were found, the magnitude of potential differences was determined using established effect size (ES) metrics. For the independent *t*-*t*ests, ES was calculated using Cohen’s *d*. Effect sizes were interpreted as trivial (< 0.20), small (0.20–0.49), moderate (0.50–0.79), and large (≥ 0.80) [35]. For the MANOVA, ES was determined using partial eta squared (η_p_^2^) with values of 0.01, 0.06, and 0.14 interpreted as small, moderate, and large, respectively [35].

## Results

Table 1 summarises the overall match-play running performance of U16 and U18 inter-county players. The mean distance covered by U16 players, irrespective of position, was 6251 ± 1394 m, corresponding to a relative intensity of 88 ± 20 m·min ^−1^. Players completed 826 ± 371 m of HSR, of which 206 ± 177 m was spent in speed zone 5 (≥ 5.5 m·s ^−1^). The number of accelerations and decelerations completed was 36 ± 11 and 44 ± 13, respectively, while the mean max speed attained during match play was 6.94 ± 0.36 m·s^-1^. For the U18 group, the mean distance completed was 6773 ± 1221m, representing a relative intensity of 93 ± 16 m·min ^−1^. Players achieved a total of 1071 ± 424 m of high-speed running, with 348 ± 195 m occurring in speed zone 5. The total number of accelerations and decelerations completed was 36 ± 12 and 48 ± 14, respectively. The average maximum speed attained was 6.94 ± 0.40 m·s^-1^.

**Table 1:** Match-play running performance of U16 and U18 LGF.

Performance Variable	U16	U18	*p*	95% CI (Diff)	Cohen’s *d*
**Distance (m)**	6251 ± 1394	6773 ± 1221	0.008*	[−906, −139]	0.40
**High Speed Running (m)**	826 ± 371	1071 ± 424	< 0.001*	[−364, −125]	0.61
**Max Speed (m·s**^**-1**^)	6.94 ± 0.36	6.94 ± 0.40	0.893	[-0.38, 0.43]	0.02
**Relative Distance (m·min**^**-1**^)	88 ± 20	93 ± 16	0.063	[−10, 0]	0.28
**Distance Speed Zone 4 (m)**	620 ± 242	723 ± 267	0.008*	[−179, −27]	0.40
**Distance Speed Zone 5 (m)**	206 ± 177	348 ± 195	< 0.001*	[−197, −86]	0.76
**Accelerations (n) (≥ 3 m·s**^**-2**^)	36 ± 11	36 ± 12	0.994	[−4, 4]	0.00
**Decelerations (n) (≤ −3 m·s**^**-2**^)	44 ± 13	48 ± 14	0.075	[−8, 0]	0.27

Data presented as mean ± SD. *Statistically significant difference between age grades (p < 0.05). Effect sizes (Cohen’s d) are interpreted as trivial (< 0.20), small (0.20–0.49), moderate (0.50–0.79), and large (≥ 0.80).

Multivariate analysis for within- and between-subjects factors indicated significant main effects for halves of play (F(9, 165) = 13.974, *p* < 0.001, η_p_^2^ = 0.431), team (F(9, 165) = 7.855, *p* < 0.001, η_p_^2^ = 0.300), and playing position (F(36, 672) = 4.187, *p* < 0.001, η_p_^2^ = 0.183) on the combined dependent variables. The interaction between team and playing position was not statistically significant for within- and between-subjects factors (F (36,672) = 0.724, *p* = 0.884, η_p_^2^ = 0.037 and F (36, 672) = 1.315, *p* = 0.106, η_p_^2^ = 0.067). The 3-way interaction between halves of play, age-grade, and position was also not significant (F (36,672) = 0.724, *p* = 0.884, η_p_^2^ = 0.037).

Table 2 summarises the follow-up univariate tests. For halves of play, the greatest effect was seen for relative distance, which saw a large decrease in the second half of play (*p* < 0.001, η_p_^2^ = 0.225). Moderate, statistically significant reductions were also found for HSR (*p* = 0.001, η_p_^2^ = 0.059) and distance covered in Zone 4 (*p* = 0.003, η_p_^2^ = 0.049). No statistically significant differences between halves of play were observed for maximum speed, accelerations or decelerations.

**Table 2:** Effects of half, team, and playing position on performance measures.

*Performance Variable*	*Source*	*F (df1, df2)*	*p*	η_p_^2^
Total Distance (m)	Half	5.89 (1, 165)	0.016*	0.033
	Team	19.38 (1, 165)	<0.001*	0.101
	Position	39.55 (4, 165)	<0.001*	0.478
High Speed Running (m)	Half	10.79 (1, 165)	0.001*	0.059
	Team	34.71 (1, 165)	<0.001*	0.167
	Position	22.50 (4, 165)	<0.001*	0.342
Max Speed (m·s^-1^)	Half	1.00 (1, 165)	0.318	0.006
	Team	0.29 (1, 165)	0.591	0.002
	Position	3.36 (4, 165)	0.011*	0.072
Relative Distance (m·min^-1^)	Half	50.30 (1, 165)	<0.001*	0.225
	Team	12.61 (1, 165)	<0.001*	0.068
	Position	46.11 (4, 165)	<0.001*	0.516
Distance Zone 4 (m)	Half	8.89 (1, 165)	0.003*	0.049
	Team	20.31 (1, 165)	<0.001*	0.105
	Position	29.50 (4, 165)	<0.001*	0.405
Distance Zone 5 (m)	Half	4.34 (1, 165)	0.039*	0.024
	Team	35.29 (1, 165)	<0.001*	0.169
	Position	7.52 (4, 165)	<0.001*	0.148
Accelerations (n) (≥ 3 m·s^-2^)	Half	0.27 (1, 165)	0.601	0.002
	Team	0.00 (1, 165)	0.998	0.000
	Position	2.09 (4, 165)	0.085	0.046
Decelerations (n) (≤ −3 m·s^-2^)	Half	0.96 (1, 165)	0.329	0.006
	Team	4.16 (1, 165)	0.043*	0.023
	Position	7.87 (4, 165)	<0.001*	0.154

*Statistically significant difference between halves of play, team or position (p < 0.05). Effect sizes (η_p_^2^) are interpreted as small (≥ 0.01), moderate (≥ 0.06), and large (≥ 0.14).

The comparison of age grades indicated that the U18 group exhibited superior running outputs compared to the U16 group across multiple variables. Significant differences were observed across age grades for high-speed running distance (*p* < 0.001, η_p_^2^ = 0.167) and the distances covered in speed zones 4 (*p* < 0.001, η_p_^2^ = 0.105) and 5 (*p* < 0.001, η_p_^2^ = 0.169). Statistically significant differences were observed between the age grades for TD (*p* < 0.001, η_p_^2^ = 0.101) and relative distance (*p* < 0.001, η_p_^2^ = 0.068). No statistically significant differences were found between the age groups regarding maximum speed or acceleration. Playing position had the greatest significant influence on nearly all performance variables, consistently demonstrating large effect sizes. Large effects were found for TD (*p* < 0.001, η_p_^2^ = 0.478), HSR (*p* < 0.001, η_p_^2^ = 0.342), relative distance (*p* < 0.001, η_p_^2^ = 0.516), distance in speed zones 4 (*p* < 0.001, η_p_^2^ = 0.405), and 5 (*p* < 0.001, η_p_^2^ = 0.148), and decelerations (*p* < 0.001, η_p_^2^ = 0.154). No statistically significant differences in the number of accelerations were observed across playing positions.

**Fig 1:**
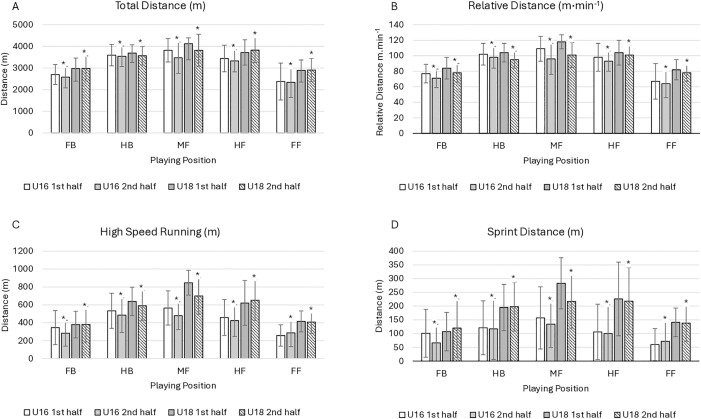
The positional running performance profile of U16 and U18 LGFA players across halves of play for (A) Total Distance (m), (B) Relative Distance (m·min^-1^) (C) High Speed Running (m; ≥ 4.4 m·s^-1^), and (D) Sprint Distance (m; ≥ 5.5 m·s^-1^). Data is reported as mean ± SD. * Significant difference (*p* < 0.05) between halves of play.

Table 3 and Fig 1 present the post hoc Bonferroni comparisons. Running outputs were consistently higher for the middle eight positions (half-backs, midfielders, half-forwards) compared to the inside lines (full-backs and full-forwards). These positions also showed the greatest decline in second-half performance, particularly in high-intensity metrics, e.g., relative distance and HSR. Higher physical outputs were observed in all positions as the age profile increased.

**Table 3:** Running performance of U16 and U18 LGF by playing position and halves of play.

Playing Position		FB	HB	MF	HF	FF
***Total Distance (m)***						
*U16*	*1st Half*	2698 ± 461^bcdx^	3594 ± 494^aex^	3820 ± 542^aex^	3440 ± 613^aex^	2376 ± 853^bcdx^
	*2nd Half*	2578 ± 500^bcdx†^	3539 ± 476^aex†^	3473 ± 719^aex†^	3330 ± 510^aex†^	2341 ± 696^bcdx†^
*U18*	*1st Half*	2973 ± 488^bcdy^	3690 ± 382^aey^	4122 ± 269^aey^	3715 ± 592^aey^	2891 ± 480^bcdy^
	*2nd Half*	2979 ± 580^bcdy†^	3567 ± 431^aey†^	3822 ± 738^aey†^	3824 ± 577^aey†^	2907 ± 541^bcdy†^
***High Speed Running (m)***						
*U16*	*1st Half*	346 ± 190^bcdx^	533 ± 196^aex^	565 ± 191^aex^	459 ± 201^aex^	258 ± 120^bcdx^
	*2nd Half*	283 ± 145^bcdx†^	486 ± 194^aex†^	479 ± 155^aex†^	424 ± 176^aex†^	287 ± 153^bcdx†^
*U18*	*1st Half*	380 ± 149^bcdy^	639 ± 158^aey^	847 ± 139^aey^	621 ± 251^aey^	415 ± 117^bcdy^
	*2nd Half*	382 ± 197^bcdy†^	591 ± 169^aey†^	699 ± 205^aey†^	652 ± 226^aey†^	408 ± 124^bcdy†^
***Max Speed (m·s*** ^***−1***^**)**						
*U16*	*1st Half*	6.97 ± 0.40^b^	7.05 ± 0.41^a^	7.10 ± 0.41	6.94 ± 0.38	6.79 ± 0.44
	*2nd Half*	6.71 ± 0.54^b^	7.12 ± 0.46^a^	6.97 ± 0.29	6.91 ± 0.36	6.70 ± 0.58
*U18*	*1st Half*	6.84 ± 0.51^b^	6.98 ± 0.47^a^	7.13 ± 0.34	6.97 ± 0.50	6.94 ± 0.39
	*2nd Half*	6.76 ± 0.63^b^	7.00 ± 0.38^a^	7.11 ± 0.51	6.92 ± 0.47	7.01 ± 0.38
***Relative Distance (m·min*** ^***-1***^ **)**						
*U16*	*1st Half*	77 ± 12^bcdx^	102 ± 14^aex^	109 ± 16^aex^	98 ± 18^aex^	67 ± 23^bcdx^
	*2nd Half*	71 ± 12^bcdx†^	98 ± 14^aex†^	96 ± 20^aex†^	93 ± 13^aex†^	64 ± 18^bcdx†^
*U18*	*1st Half*	84 ± 14^bcdy^	104 ± 12^aey^	118 ± 9^aey^	104 ± 16^aey^	82 ± 13^bcdy^
	*2nd Half*	78 ± 12^bcdy†^	95 ± 11^aey†^	101 ± 16^aey†^	101 ± 12^aey†^	78 ± 11^bcdy†^
***Distance Speed Zone 4 (m)***						
*U16*	*1st Half*	245 ± 121^bcdx^	411 ± 130^aex^	408 ± 102^aex^	354 ± 127^aex^	198 ± 82^bcdx^
	*2nd Half*	217 ± 94^bcdx†^	369 ± 114^aex†^	345 ± 103^aex†^	324 ± 121^aex†^	215 ± 109^bcdx†^
*U18*	*1st Half*	273 ± 98^bcdy^	445 ± 95^aey^	563 ± 107^aey^	395 ± 152^aey^	274 ± 91^bcdy^
	*2nd Half*	262 ± 116^bcdy†^	393 ± 112^aey†^	483 ± 145^aey†^	434 ± 138^aey†^	270 ± 73^bcdy†^
***Distance Speed Zone 5 (m)***						
*U16*	*1st Half*	101 ± 87^bcdx^	121 ± 98^aex^	157 ± 113^aex^	106 ± 101^aex^	60 ± 58^bcdx^
	*2nd Half*	66 ± 68^bcdx†^	117 ± 111^aex†^	134 ± 85^aex†^	100 ± 105^aex†^	72 ± 73^bcdx†^
*U18*	*1st Half*	107 ± 70^bcdy^	195 ± 84^aey^	283 ± 93^aey^	226 ± 134^aey^	141 ± 52^bcdy^
	*2nd Half*	120 ± 106^bcdy†^	198 ± 89^aey†^	217 ± 97^aey†^	218 ± 122^aey†^	138 ± 66^bcdy†^
***Accelerations (n) (≥ 3 m·s*** ^***-2***^ **)**						
*U16*	*1st Half*	19 ± 7	19 ± 6	21 ± 6	18 ± 6	15 ± 6
	*2nd Half*	17 ± 8	18 ± 4	19 ± 6	20 ± 5	14 ± 7
*U18*	*1st Half*	17 ± 8	21 ± 6	18 ± 7	17 ± 6	17 ± 6
	*2nd Half*	18 ± 8	20 ± 7	19 ± 10	19 ± 5	16 ± 5
***Decelerations (n) (≤ −3 m·s*** ^***-2***^ **)**						
*U16*	*1st Half*	22 ± 7^x^	26 ± 6^ex^	23 ± 6^ex^	22 ± 6^x^	16 ± 8^bcx^
	*2nd Half*	20 ± 7^x^	25 ± 7^ex^	22 ± 7^ex^	23 ± 8^x^	16 ± 9^bcx^
*U18*	*1st Half*	23 ± 8^y^	28 ± 7^ey^	25 ± 4^ey^	23 ± 7^y^	20 ± 6^bcy^
	*2nd Half*	24 ± 9^y^	27 ± 7^ey^	24 ± 10^ey^	23 ± 8^y^	19 ± 5^bcy^

Data reported as mean ± SD. a: significantly different (p < 0.05) from FB; b: significantly different (p < 0.05) from HB; c: significantly different (p < 0.05) from MF; d: significantly different (p < 0.05) from HF; e: significantly different (p < 0.05) from FF; xy: significant difference (p < 0.05) between teams; †: significant difference (p < 0.05) between halves of play.

## Discussion

This study is the first to detail the GPS-derived running demands of U16 and U18 inter-county Ladies’ Gaelic football by playing position and halves of play. It was hypothesised that the running performance profile would differ between halves and playing position at each age grade and that U18 players would exhibit greater performance demands than U16 players. The main findings reveal a decline in running performance across halves of play, regardless of playing position and age grade. The middle eight positions exhibit the highest match-play demands and present a distinct positional running profile compared with the inside lines, while U18 players cover considerably greater total and high-speed running distances than U16 players.

Independent of playing position, U18 players demonstrated significantly greater total distance (*p* = 0.008), HSR (*p* < 0.001), and distance in speed zones 4 (*p* = 0.008) and 5 (*p* < 0.001) than U16 players, with small-to-moderate effect sizes. However, while there were no statistically significant differences between age grades for overall relative distance (*p* = 0.063), maximum speed (*p* = 0.893), accelerations (*p* = 0.994), or decelerations (*p* = 0.075), positional breakdowns revealed a more nuanced profile. Specifically, when controlling for playing position, U18 players demonstrated significantly higher relative distances than their U16 counterparts across multiple positional lines (Table 3). The mean total distance covered by U16 players was 6251 ± 1394 m while that of U18’s was 6773 ± 1221 m, corresponding to a relative distance of 88 ± 20 m·min^-1^ and 93 ± 16 m·min^-1^, respectively. For the U16 group, 13% of the total distance was spent in high-speed running and 3% in sprinting, rising to 16% and 5%, respectively, for the U18 group. The transition from U16 to U18, therefore, involves not only an increase in overall running volume (total distance) but also a greater accumulation of high-intensity efforts, as evidenced by the increased high-speed running and distance covered in speed zones 4 and 5. Consequently, coaches should progressively increase the emphasis on high-intensity conditioning to effectively prepare players for the demands of U18 and subsequently senior match-play [28].

The frequency of both accelerations and decelerations was statistically‌‌ similar across groups (*p* > 0.05), indicating that the fundamental requirement for initiating rapid speed changes and effective braking is comparable between U16 and U18 levels. However, given the high absolute frequency of these mechanically demanding actions, coaches should incorporate targeted deceleration training [36] alongside multi-directional agility drills that focus on controlled stops and directional changes at high speeds [37]. These movement capacities can then be progressively applied within sport-specific contexts [37]. Implementing these strategies, in conjunction with neuromuscular power development, prepares players for the highly intermittent nature of the sport, which requires frequent transitions between low-intensity movements and high-intensity, multi-directional efforts [8].

No significant differences were found between the groups for maximal speed (U16: 6.94 ± 0.36; U18: 6.94 ± 0.40 m·s^-1^), consistent with previous research indicating a plateau in sprint performance after mid-adolescence [28,38,39]. Investigations in soccer players found that no significant improvement in sprinting performance over 18.2 m occurred after age 14 [39]. Similarly, no significant differences in 20 m sprinting speed have been found between U17 and U19 Irish international soccer players, or between U15, U17, and U20 Brazilian international soccer players [28,38]. Analysis of U14, U16 and U18 inter-county Ladies’ Gaelic footballers also found no significant difference in max speed over 20 m [3].

However, evidence suggests this trend can be altered through targeted physical preparation. Research in younger female athletes (~13 years of age) demonstrates that targeted strength and plyometric training can significantly enhance sprinting performance [40]. This trainability extends into late adolescence, where structured strength and plyometric interventions have been shown to significantly improve linear sprint speed in adolescent female team sport athletes, with strength training yielding particularly strong adaptations in those older than 15 years [41,42]. Therefore, the plateau observed in the U16 and U18 cohorts may reflect a lack of consistent, targeted physical development, highlighting the need for progressive S&C programs to drive continued speed adaptations. Currently, neither the mean nor the range of sprint distances has been described in Ladies’ Gaelic football. It may be that players do not achieve ‘true’ maximum sprinting speeds during match-play, as the sprint distances are insufficient due to the intermittent nature of the game. However, in elite inter-county Ladies’ Gaelic football, peak speeds of 86 ± 4% were achieved across all positions, compared with peak speeds obtained during a 40 m linear sprint test in a pre-season testing battery [6,43].

A primary contribution of this research is the establishment of the first positional benchmarks across U16 and U18 age grades in LGF. To facilitate this objective tracking of developmental progress towards established senior standards [34], standard absolute velocity thresholds were employed, consistent with recent research on female and youth cohorts [2,6,24,28]. However, when applying fixed, arbitrary criteria, consideration should be given to the inherent methodological limitations [44,45]. While useful for standardised comparison, determining ‘where to draw the lines’ in female codes often lacks population-specific accuracy [45], and small variations in threshold definitions can considerably alter reported match demands [44]. A fundamental limitation is that fixed thresholds solely quantify external playing load (GPS-derived distance) without accounting for individual physical capacities such as anaerobic fitness or maximum velocity [45]. Therefore, while these newly established LGF benchmarks provide collective profiles useful for broad benchmarking, they may obscure individual differences. The same absolute speed might represent a near-maximal effort for one player but only moderate intensity for another, reflecting vastly different internal workloads (physiological stress) among individual athletes within the same positional group [44,45].

Developmental differences have been identified in several other female team sports, such as soccer, AFLW, and field hockey, as well as at the youth level in male Gaelic games [3,4,7,13,14,24,46,47]. Among youth soccer players, notable differences in work rates were observed between U15, U16, and U17 players [15,24]. The total, high-intensity (4.3–5.5 m·s^-1^), and sprinting (≥ 5.5 m·s^-1^) distances covered by U15 players were all lower than those recorded for U16 and U17 cohorts [24]. These age-related differences remained even after adjusting for match duration, with relative distances of 86 m·min^-1^ reported for U15’s compared to 93 and 95 m·min^-1^ for U16 and U17 players [24]. Similarly, in international-level field hockey, relative intensity, HSR (4.4–5.5 m·s^-1^) and sprinting distances (≥ 5.5 m·s^-1^) increased between the U17 and U21 age grades [48]. Collectively, these findings suggest a clear developmental progression in physical capacity across age groups as players mature. However, direct comparison between these studies and the current data remains challenging due to sport-specific rules, context, pitch sizes, playing numbers, differing research methodologies, and the lack of standardisation regarding GPS hardware models and velocity thresholds used across different sports [2,11,34].

The consistent pattern of increasing demands across various team sports highlights the need to implement evidence-informed training structures specifically aligned with the increasing physical requirements of Ladies’ Gaelic football as young athletes mature. Regular exposure to targeted, high-intensity conditioning stimuli is fundamental to facilitating the unique physiological and neuromuscular adaptations that underpin these increased physical capacities [49]. By quantifying these competitive requirements, coaches can establish a structured, periodised approach that combines isolated onditioning for targeted physical overload, such as sprint interval training (SIT) or repeated sprint ability, with game-like scenarios (e.g., SSGs) to prepare players for match situations [49]. Practitioners can adjust SSG constraints, such as using larger relative pitch sizes with fewer players or incorporating specific rules (e.g., requiring sprints after passing), to develop in-game decision-making alongside anaerobic power [8,50]. Moreover, prioritising these repeated conditioning stimuli and ensuring specific overload within a technical and tactical context is essential for developing game-specific fitness and preparing players for the ‘worst-case scenario’ intensities encountered at each developmental stage [49]. Therefore, as players move from U14 to U16 and U18 levels, coaches should progressively increase the emphasis on these targeted, multifaceted conditioning strategies to support the ongoing adaptations in speed and high-intensity running required for senior performance.

The large effect sizes for TD (*p* < 0.001, η_p_^2^ = 0.478), HSR (*p* < 0.001, η_p_^2^ = 0.342), relative distance (*p* < 0.001, η_p_^2^ = 0.516), and distance in speed zone 4 (*p* < 0.001, η_p_^2^ = 0.405) and 5 (*p* < 0.001, η_p_^2^ = 0.148) underscore that playing position is the most influential factor dictating player workload in Ladies’ Gaelic Football. Midfielders, half-backs, and half-forwards consistently demonstrated the highest outputs, likely due to their pivotal tactical role linking offensive and defensive transitions [6]. Conversely, the ‘inside’ full-back and full-forward lines covered significantly less total and high-speed running, reinforcing their more specialised roles involving explosive, short movements within tighter areas [2,6]. This is consistent with observations in other adolescent female invasion sports and senior intercounty LGF [2,6,13,18,21,39]. The consistency in positional demand patterns across U16, U18, and senior levels suggests that tactical roles and associated physical requirements are established early in a player’s development.

Logistical constraints, such as limited contact time, are common in amateur and youth settings [5]. To maximise the limited contact time available, practitioners should prioritise structured SSGs as an efficient, integrated training modality that addresses physical, technical, and tactical requirements simultaneously [51]. This integrated approach aligns with contemporary tactical periodisation models for Gaelic football, which advocate for embedding conditioning elements directly within tactical practice [51]. For example, coaches can utilise larger SSGs with relative pitch sizes to optimise high-speed running, while incorporating smaller grids targeting high-intensity, explosive actions and anaerobic power [52]. This ensures players are prepared for both transitional running demands and high-intensity, localised engagement, regardless of their playing position. Furthermore, if second-half fatigue is identified as a limiting factor, coaches can efficiently implement SSG constraints (e.g., high-press rules) at the end of practice to ensure players develop the capacity to maintain technical and tactical proficiency under intense physical load [48].

While providing targeted preparation is necessary, coaches should also balance positional demands with principles of long-term player development. Youth players should be encouraged to experience different playing positions during training and games to develop a broad technical skill set and overall tactical awareness [53]. By avoiding early positional specialisation, players are forced to solve varied technical and tactical problems from different perspectives (e.g., viewing an overlap as both a defender and an attacker). This constant exploration and adaptation to varied constraints is essential for developing the deep ‘gamesense’ and adaptability required in adult competition [53]. Concurrently, early physical conditioning should focus on developing general athletic capacities before progressively shifting toward more position-specific qualities [5]. Developing this broad physical foundation is beneficial as positional roles are often interchangeable throughout the entire developmental pathway (U16, U18, and senior) [54]. Consequently, ensuring all players possess a robust physical capacity prepares them to meet the high running demands observed in ‘middle eight’ positions, providing tactical flexibility for the coach at all age grades [54].

## Limitations

This study is not without its limitations. While data was collected over a two-year period, the number of competitive matches played by both teams, and thus the sample size, was relatively low (U16: n = 9; U18, n = 12). The study was conducted in a single county with a large playing population, and these results may be more generalisable to similar counties and not to those with smaller playing populations that also compete in the same championship. Training methods and philosophies, approaches to long-term player development, and competition can vary across counties and provinces, which may limit the representativeness of these results to the broader underage LGF landscape. Secondly, the specific match context, such as game importance, time of year, opposition quality, surface type, or match outcome (win/loss), which is known to significantly affect player work rates and tactical approaches, was not analysed [21,47,48]. Thirdly, Horizontal Dilution of Precision (HDOP) data was not reported. HDOP is an indicator of GPS signal strength and positional accuracy. Therefore, the precision and potential variability in data accuracy across different matches and locations remain unknown, which could impact the reliability of the measured data [11]. Fourthly, the data collection approach, where individual player samples were collected between 1 and 6 times per player, introduces potential variability. While providing a broad dataset, this uneven sampling might not fully capture the typical performance profile of individual players over a season and could potentially bias findings if players with more data points differ systematically from those with fewer. Finally, we acknowledge that while direct validation of the specific ‘Catapult One’ branding has not been reported, as detailed in our methodology, this concern is mitigated because the device utilises established PlayerTek 10-Hz technology, which is validated and highly reliable within inter-county Gaelic football contexts [9,33].

## Practical applications

The findings of this investigation have important and direct implications for coaches and practitioners working with female youth athletes in Gaelic football. This foundational data addresses the current reliance on anecdotal evidence or generic data from other sports or male populations, providing coaches with female-specific benchmarks essential for appropriate and effective training [3,29]. Coaches can utilise this information to design evidence-informed training programmes that progressively increase the volume and intensity of high-speed running, sprinting, and explosive actions to prepare U16 players for the greater physical demands of the U18 age group, and U18 players for the requirements of senior competition. The identified distinct locomotor profiles across positional lines suggest that training should be tailored to these specific demands while maintaining a balanced approach to long-term athletic development. The high frequency of accelerations and decelerations highlights the sport’s intermittent nature, indicating that coaches should incorporate targeted activities to develop explosive performance and reduce injury risk. By providing detailed positional and age-grade performance profiles, this research serves as a tool for talent identification, individual player monitoring, and the establishment of clear developmental progression within the player pathway.

## Conclusion

This study offers the first comprehensive GPS-derived insights into the match-play running demands of U16 and U18 inter-county Ladies’ Gaelic football, contributing important new knowledge to a significantly under-researched area. The findings highlight significant developmental and positional differences and show a decline in running performance across halves of play. Future research should extend these insights through longitudinal studies that monitor individual athlete development, assess the effectiveness of targeted training interventions to reduce second-half performance declines, examine the impact of contextual factors on match-play running performance, and explore the complex interactions among physical, technical, and tactical execution in inter-county Ladies’ Gaelic football.

## Supporting information

S1 Data Full match running performance data.
This file contains the anonymised GPS metrics for U16 and U18 players for the duration of the full match.
(XLSX)

S2 Data Halves of play running performance data.
This file contains the anonymised GPS metrics for U16 and U18 players broken down by first and second halves. https://doi.org/10.17605/OSF.IO/SE2MK.
(XLSX)
